# IL-3 and CSF-1 Interact to Promote Generation of CD11c^+^ IL-10-Producing Macrophages

**DOI:** 10.1371/journal.pone.0095208

**Published:** 2014-04-17

**Authors:** Kuo-Ching Sheng, Lara J. Herrero, Adam Taylor, Andrew J. Hapel, Suresh Mahalingam

**Affiliations:** 1 Institute for Glycomics, Griffith University, Gold Coast Campus, QLD, Australia; 2 Division of Molecular Medicine, The John Curtin School of Medical Research, The Australian National University, ACT, Australia; University of Georgia, United States of America

## Abstract

Unraveling the mechanisms of hematopoiesis regulated by multiple cytokines remains a challenge in hematology. IL-3 is an allergic cytokine with the multilineage potential, while CSF-1 is produced in the steady state with restricted lineage coverage. Here, we uncovered an instructive role of CSF-1 in IL-3-mediated hematopoiesis. CSF-1 significantly promoted IL-3-driven CD11c^+^ cell expansion and dampened basophil and mast cell generation from C57BL/6 bone marrow. Further studies indicated that the CSF-1/CSF-1R axis contributed significantly to IL-3-induced CD11c^+^ cell generation through enhancing c-Fos-associated monopoiesis. CD11c^+^ cells induced by IL-3 or IL-3/CSF-1 were competent in cellular maturation and endocytosis. Both IL-3 and IL-3/CSF-1 cells lacked classical dendritic cell appearance and resembled macrophages in morphology. Both populations produced a high level of IL-10, in addition to IL-1, IL-6 and TNFα, in response to LPS, and were relatively poor T cell stimulators. Collectively, these findings reveal a role for CSF-1 in mediating the IL-3 hematopoietic pathway through monopoiesis, which regulates expansion of CD11c^+^ macrophages.

## Introduction

Interleukin-3 (IL-3) is a hematopoietic cytokine that is secreted by activated T and mast cells (MCs) [1], [2], [3]. It is produced during allergic inflammations or in response to parasitic infections, and has been considered a Th2 response promoter [3], [4]. IL-3 is necessary for optimal immunity against helminthes, reflecting its role in enhancing generation of systemic basophils and tissue MCs [5]. The hallmark of IL-3 is its capacity to stimulate proliferation of pluripotent hematopoietic stem cells and progenitor cells, particularly those of the myeloid lineage, at various developmental stages [6], [7]. As such, IL-3 has been recognized as a multi-potential colony-stimulating factor (multi-CSF) [7], [8]. IL-3 stimulates *ex vivo* generation of MCs, macrophages, basophils and CD11c^+^ cells in mouse bone marrow (BM) [9], [10]. The hematopoietic effect of IL-3 has been shown to be strain-specific [11]. In contrast to IL-3, CSF-1 or macrophage colony-stimulating factor (M-CSF) is produced in the steady state, with a much more restricted hematopoietic coverage, primarily regulating development of the monocyte lineage [12], [13], [14], and has been reported to have a role in developing dendritic cells (DCs) [15].

The capacity of IL-3 to promote multilineage development has been examined in conjunction with several cytokines. For instance, IL-3, IL-6 and stem cell factor mediate generation of granulocytes and monocytes, and this combination has been used to study myeloid lineage commitment of C57BL/6 mice [16], [17]. Together with TNFα, IL-3 is a potent cytokine in generation of Langerhans cells from human cord blood CD34^+^ hematopoetic progenitor cells (HPCs) [18]. In humans, IFNγ cooperates with IL-3 to enhance expansion of HPCs [19], while IFNβ and IL-3 induce monocyte differentiation into DCs, which potently stimulate helper T cells [20]. IL-4 and IL-3 initiate human monocyte differentiation into Th2-polarizing DCs [21]. While IL-3 and CSF-1 were known to act synergistically in induction of BM colonies and CSF-1R^+^ hematopoietic cells [22], [23], their hematopoietic relationship has not been fully characterized. With advanced knowledge in leukocyte biology and cellular/molecular techniques, we revisited earlier studies on IL-3 hematopoiesis and its lineage relationship with CSF-1 and addressed issues pertinent to a) phenotypic identifications of leukocyte populations induced by IL-3 with and without CSF-1 in BM *ex vivo*, b) the effect of CSF-1 in IL-3 multilineage hematopoiesis and associated molecular pathways and c) clarification of macrophage or DC induction as previously reported and their immunological functions. We identified an instructive role of CSF-1 in IL-3-mediated hematopoiesis and demonstrated that the combination of the two cytokines stimulated expansion of CD11c^+^ macrophages that produced IL-10 with a minimal T cell stimulation capacity.

## Materials and Methods

### Animals

C57BL/6 and Balb/c mice (aged 6–10 wk) used throughout this study were purchased from the Animal Resources Centre (Canning Vale, Western Australia). These mice, together with Csf1r-EGFP (MacGreen) [24] derived from the University of Queensland, were maintained in the animal facilities of Griffith University (Gold Coast, Australia). All experimental procedures were conducted following the animal ethics guideline of Griffith University Animal Ethics Committee (GLY/06/12/AEC).

### Bone marrow culture

BM cells from murine femurs and tibias were collected and treated with red blood cell lysis buffer (BD Biosciences, Franklin Lakes, NJ) for 5 min. Cells were washed and cultured with complete RPMI 1640 media at 5×10^5^ cells/ml in 24-well plates. Recombinant mouse IL-3 (eBioscience, San Diego, CA), GM-CSF and CSF-1 (eBioscience) were used at 10 ng/ml. At days 3, 6 and 9, one half of the culture media was aspirated and slowly replenished with media containing 20 ng/ml cytokines, without disturbing cells as described [25]. In some experiments, cells were co-incubated with 5 µg/ml CSF-1R blocking antibody anti-CD115 (AFS98) (eBioscience) [26] or isotype control antibody (Rat IgG2a κ) (eBioscience).

### Cell phenotype and morphology

Cells (2×10^5^) were pelleted and treated with Fc Block (2.4G2, BD Biosciences). They were then labeled with fluorescent antibodies including FITC-anti-CD11b (M1/70, BD), APC-eflour780-anti-CD11c, APC-anti-FcεRI (MAR-1, eBiosceince) and Percp-anti-c-kit (2B8, Biolegend). The cell/antibody mix was incubated at 4°C for 30 min. Cells were washed for flow cytometry analysis (CyAn ADP, Beckman Coulter). To examine cell morphology, cells were imaged by the 40X lens in bright field in a 24-well plate, using the ECLIPSE TS100 microscope (Nikon, Tokyo, Japan).

### Purification of CD11c^+^ and T cells

CD11c^+^ and T cells were purified by magnetic cell sorting. To purify CD11c^+^ cells, BM culture cells were incubated with anti-CD11c (N418) MAC beads (10 µl/10^7^ cells) (Miltenyi Biotec, Auburn, CA) in the presence of 2% FCS and 2 mM EDTA in PBS at 4°C for 15 min. Cells were positively selected with the MAC LS column and the magnetic separator (Miltenyi Biotech). CD11c^+^ cell purity was at least 93%. T cells were isolated from spleen. Briefly, splenocytes were incubated with a cocktail of biotin-conjugated antibodies and anti-biotin microbeads (Miltenyi Biotech) to label non-T cells. Cells were negatively selected with the LS column as described. T cell purity was >90%.

### Cytokine production

CD11c^+^ cells (10^6^/ml) were seeded in triplicates in the 24-well plates and stimulated by LPS (1 µg/ml) for 24 h. Supernatants were collected for cytokine detection by ELISA. TNFα, INFγ, IL-1β and IL-6 were detected using BD Biosciences kits, while IL-10, R&D Systems kits. Briefly, antibody-coated microtiter plates (Sarstedt, Nümbrecht, Germany) were washed with 0.05% tween in PBS, and samples were added after blocking with FCS for 2 h. Plates were washed and biotin coated detection antibody added for 2 h. Streptavidin-horseradish-peroxidase and the substrate (R&D) were added and the colorimetric reaction was stopped by 2N H_2_SO_4_. The optical density of each well was determined at 450 nm by Wallac Vitor3 1420 Multi-label Counter (PerkinElmer, Waltham, MA).

### CD11c^+^ cell maturation and antigen uptake

Cells were stimulated with LPS (1 µg/ml) derived from *Escherichia coli* (0111:B4; Sigma-Aldrich, St. Louis, MO) at 37°C for 24 h. Cells (5×10^5^) were collected, washed and incubated with Fc Block at 4°C for 5 min. They were labeled with APC-conjugated anti-CD40 (HM40-3, eBioscience), FITC-anti-CD80 (16-10.A1, eBioscience), PE-Cy7-anti-CD86 (GL1, BD), APC-MHC-class I (H2Kb) (AF6-88.5.5.3, eBioscience) and PE-Cy7-MHC class II (IA, IE) (M5/114.15.2, Biolegend, San Diego, CA), together with APC-anti-eFlour780-anti-CD11c (N418, eBioscience) or PE-anti-CD11c (HL3, BD) at 4°C for 30 min. In flow cytometry, live CD11c^+^ PI-negative cells were gated to evaluate maturation. To evaluate antigen uptake, cells were incubated with 10 µg/ml FITC-dextran (Sigma Aldrich) at 4°C or 37°C for 15 min. Cells were then labeled with APC-eFlour780-anti-CD11c. The capacity of CD11c^+^ cells in dextran uptake was evaluated.

### Transcriptional analysis of hematopoiesis

BM cell culture was harvested at day 3 and lineage-depleted. Briefly, cells were pelleted and incubated with biotin-lineage antibody cocktail (10 µl/10^7^ cells) (Miltenyi Biotec), followed by anti-biotin MicroBeads (Miltenyi Biotec). Cells were negatively selected using LS column as described. Lineage-negative cells were collected and preserved in TRIzol (Invitrogen, Victoria, Australia) at -80°C. Total RNA was isolated. RNA (1 µg) was reverse transcribed using random primers (Promega, Madison, WI) and M-MLV Reverse Transcriptase (Promega). cDNA (50 ng) was used as a template for SYBR Green real-time PCR (Roche, NSW, Australia) on a CFX96 Touch System (Biorad, Hercules, CA) using a standard three-step melt program (95°C for 15 s, 55°C for 30 s and 72°C for 30 s). Primers were designed for the quantification of C/EBPA (F 5 CGG TGC GCA AGA GCC GAG AT; R 5 CCC GCA GCG TGT CCA GTT CA) and *c-fos* (F 5 ACT AGA GAC GGA CAG ATC TG; R 5 ATA ACG GGA ACG CAG CAG TA). Data were normalized to the housekeeping gene HPRT1 (QuantiTec, Qiagen, Victoria, Australia) and presented as the relative level of expression.

### Mixed lymphocyte reaction

Purified C57BL/6 CD11c^+^ cells (2–8×10^3^) derived from IL-3, IL-3/CSF-1 and GM-CSF culture were co-cultured in triplicates with purified 2×10^4^ Balb/c T cells, which had been labeled with 5 µM CFSE, in a 96-well round bottom plate. At day 3, cells were collected and T cell proliferation was evaluated by CFSE fluorescence in flow cytometry. The percentages of cells proliferated were determined by gating on CD3^+^ cell populations with descended FITC fluorescence.

### Statistical analysis

All data are shown as the mean ± SEM, where statistical analysis is required. Significance between experimental groups was determined by the *p* value (<0.05), using student *t* test or one-way ANOVA.

## Results and Discussion

### CSF-1 modulates the IL-3 multilineage hematopoiesis in BM

**Figure 1:**
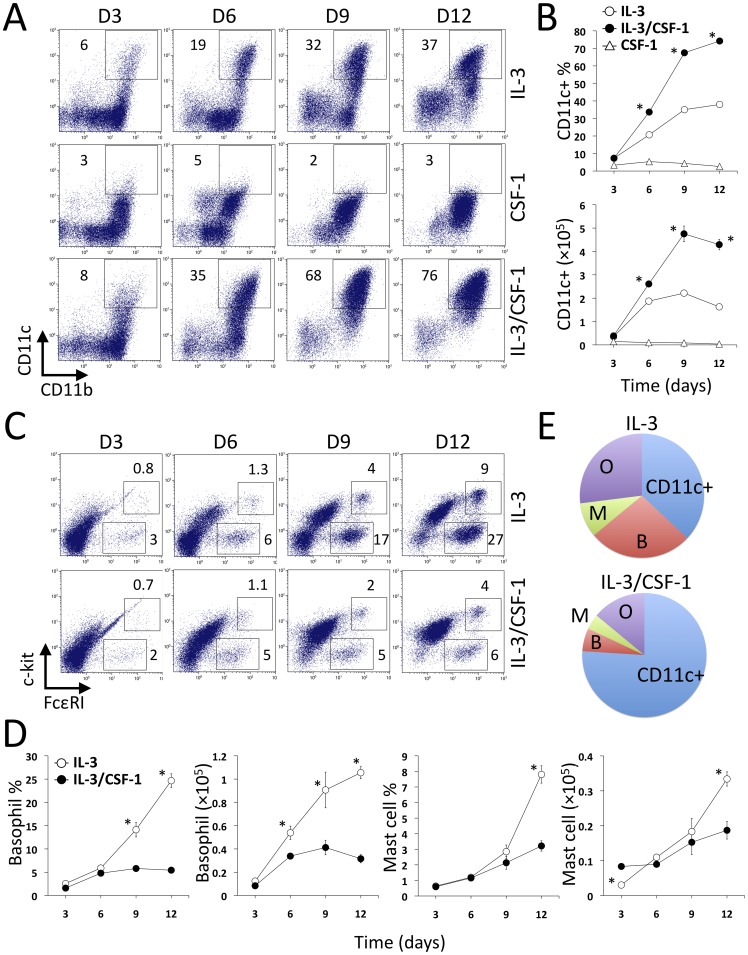
CSF-1 reinstructs IL-3-mediated hematopoiesis. BM cells were cultured with IL-3, CSF-1 or IL-3/CSF-1 in triplicates in the 24-well plate. At days 3, 6, 9 and 12, cells in each well were collected and counted, labeled with specified antibodies and analyzed by flow cytometry. The percentage and number of live CD11b^+^CD11c^+^ cells (A, B) and FcεRI^+^c-kit^–^ basophils and FcεRI^+^c-kit^+^ MCs (C, D) were determined as the mean ± SEM. Cellular compositions of IL-3 and IL-3/CSF-1 cultures at day 12 were compared in the pi chart. B, basophils; M, mast cells; O, other cells (E). Data shown are representative of four separate experiments. *p<0.05 (IL-3 vs. IL-3/CSF-1), One-way ANOVA Bonferroni test for (B) and unpaired student's t test for (D).

We have examined the effect of IL-3 on cell generation kinetics of BM *ex vivo*. IL-3 primarily induced three cell populations, CD11b^+^CD11c^+^ cells, FcεRI^+^c-kit^–^ basophils and FcεRI^+^c-kit^+^ MCs (Fig. 1). The rapid increase in numbers of CD11c^+^ cells was followed by moderate expansion of basophils, whereas the development of MCs occurred later (Fig. 1). This sequential induction of three distinct IL-3 regulated populations may be linked to their specific roles at various stages of the allergic immune response. While CSF-1 alone had a minimal effect on DC generation, the addition of CSF-1 into IL-3 treated cultures significantly increased both the percentage and absolute number of CD11c^+^ cells, with the percentage peaking at day 12 and absolute number at day 9 (Fig. 1A and 1B). CSF-1/IL-3 induced more than a two-fold increase of the CD11c^+^ cell yield, compared with IL-3 treatment alone (Fig. 1A and 1B). In contrast to CD11c^+^ cells, IL-3-induced generation of basophils and MCs, both in percentages and numbers, was depressed by the addition of CSF-1 (Fig. 1C and 1D). Both IL-3 and IL-3/CSF-1 had a comparable potent effect in inducing overall cell expansion (Fig. S1). There was no effect of CSF-1 on expansion of CD11c^+^ cells with GM-CSF (Fig. S2A), a potent cytokine that is associated with the generation of inflammatory DCs [27], even though CSF-1 was able to enhance generation of total cells in cultures containing this cytokine (Fig. S2B).

### CSF-1/CSF-1R interactions contribute to IL-3-induced CD11c^+^ cell generation by enhancing monopoiesis

**Figure 2:**
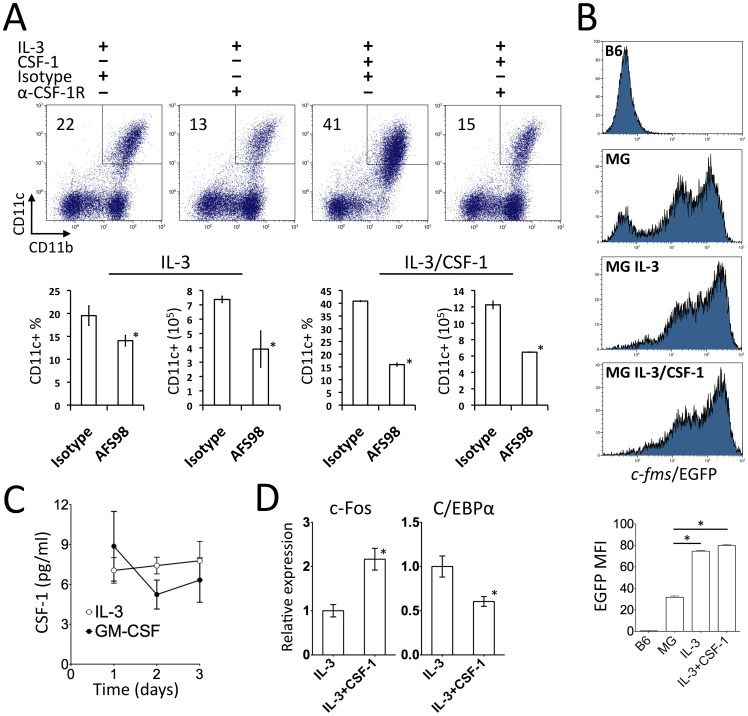
The CSF-1/CSF-1R axis contributes to IL-3-mediated CD11c^+^ cell expansion. (A) BM cells were cultured with IL-3 or IL-3/CSF-1, in the presence of the anti-CSF-1R (AFS98) or isotype control antibody in triplicates in the 24 well plate. At day 6, cells were labeled for flow cytometry analysis. The percentages and numbers of live CD11b^+^CD11c^+^ DCs derived from different conditions were compared. (B) BM cells from C57BL/6 or MacGreen (MG) were isolated and incubated with media, IL-3 or IL-3/CSF-1 in triplicates in the 24 well plate. At 24 h, cells were collected and analyzed for EGFP expression. (C) BM cells were cultured with IL-3 or GM-CSF in triplicates for 3 days. Supernatants were collected to determine CSF-1. (D) BM cells were cultured with IL-3 or IL-3/CSF-1 in triplicates. At day 3, cells were collected and lineage-depleted. Total RNA was extracted and relative expressions of c-Fos and C/EBPα transcripts were determined by real time PCR. Data shown are representative of three separate experiments. *p<0.05, One-way ANOVA Bonferroni test for (B) and student's t test, unpaired for (A) and (D).

We further investigated the mechanism by which CSF-1 modulates the IL-3-mediated hematopoietic pathway. CD11c^+^ cell generation mediated by IL-3 was significantly inhibited when CSF-1R was blocked with AFS98 (Fig. 2A). In contrast, the AFS98 antibody had no effect on the generation of CD11c^+^ cells induced by GM-CSF (Fig S3). In a positive control, AFS98 blocked the action of CSF-1, resulting in a similar level of CD11c^+^ cell generation to that seen in the AFS98 treated IL-3 culture (Fig. 2A). The dependence of IL-3 on CSF-1R in inducing CD11c^+^ generation was further validated by two findings. First, expression of the CSF-1R reporter *c-fms*/EGFP in MacGreen BM cells was rapidly induced by IL-3 with or without CSF-1 (Fig. 2B). Second, albeit at a low level, CSF-1 was produced and remained at a constant level at the early stage of IL-3 culture (Fig. 2C), while IL-34, another cytokine known to signal through CSF-1R, was not detected (data not shown). We next examined how CSF-1 transcriptionally regulates IL-3 induction of CD11c+ cells. Monopoiesis and granulopoiesis have been linked to transcriptional factors c-Fos and C/EBPα, respectively [16], [17], [28]. Indeed, in early lineage-depleted progenitor cells, CSF-1 significantly enhanced expression of transcriptional factor c-Fos, whereas it reduced expression of C/EBPα (Fig. 2D) [16]. IL-3 stimulates JAK/STAT, which activates and upregulates c-Fos and cell proliferation [29]. CSF-1 preferentially activates ERK and induces expression of c-Fos that enhances monopoiesis [17], [28]. Thus, CSF-1 skews IL-3-mediated hematopoiesis towards monopoiesis through reinforcing c-Fos, promoting differentiation of the CD11c^+^ monocytic pool [16], [17].

### Phenotypic and functional comparison between IL-3 and IL-3/CSF-1 CD11c^+^ cells

**Figure 3:**
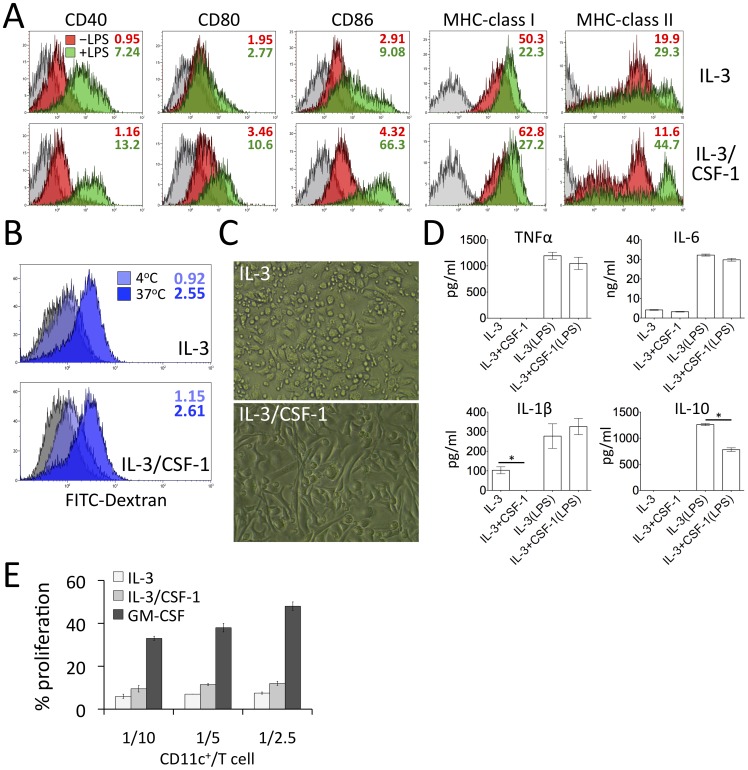
Functional comparison between IL-3 and IL-3/CSF-1 DCs. BM cells were cultured with IL-3 or IL-3/CSF-1. At day 6, CD11c^+^ cell functions were examined. (A) Isolated CD11c^+^ cells were stimulated with LPS for 24 h. The maturation state was evaluated using fluorochrome-conjugated antibodies targeting CD40, CD80, CD86, MHC-class I and II. The shaded area represents cells treated with the isotype antibody. The green and red areas represent cells with and without LPS stimulation, respectively. The numbers in colors denote respective MFI. (B) Cells were treated with 1 µg/ml FITC-dextran at 4°C for 30 min or 37°C for 15 min. The binding and uptake of dextran were determined by FITC fluorescence in flow cytometry. The shaded area represents cells only, whereas the light and bright blue areas represent cells incubated with FITC-dextran at 4°C and 37°C, respectively. The numbers in colors denote respective MFI. (C) The morphology of IL-3 and IL-3/CSF-1-induced CD11c^+^ cell culture was examined under the microscope. (D) Purified IL-3 or IL-3/CSF-1 DCs (2×10^5^/200 µl) were stimulated with LPS (1 µg/ml) in a 96-well plate. At 24 h, supernatants were collected and cytokine levels were determined by ELISA. (E) C57BL/6 CD11c^+^ cells derived from IL-3, IL-3/CSF-1 and GM-CSF BM culture were co-incubated with Balb/c CFSE-labeled T cells in specified (CD11c^+^/T cell) ratios in triplicates in the 96 well plate. Proliferation of T cells was determined and compared, based on the gate of the CD3^+^ cell population with descended CFSE fluorescence. Data shown are representative of at least three separate experiments. *p<0.05 (student's t test, unpaired).

We next compared the maturation phenotype and functions of IL-3 and IL-3/CSF-1 CD11c^+^ cells. Phenotypically, both populations expressed similar levels of costimulatory molecules CD40, CD80, CD86 and MHC-class I (H2Kb) and II (IA, IE) (Fig. 3A). After LPS stimulation, IL-3/CSF-1 CD11c^+^ cells exhibited a more mature phenotype, expressing higher levels of co-stimulatory markers, particularly CD86 (Fig. 3A). Both populations were comparable in their abilities to take up antigens based on FITC-dextran binding at 4°C and internalization at 37°C for 15 min (Fig. 3B). After 7 days of culture, both cell populations were adherent; however, IL-3/CSF-1 cells were more elongated/spreading in appearance resembling macrophages (Fig. 3C). Neither of the cell populations exhibited the conventional DC morphology. In response to LPS, both CD11c^+^ purified populations produced comparable levels of TNFα, IL-1β and a relatively high level of IL-6, (Fig. 3D). Both produced IL-10, with IL-3/CSF-1 CD11c^+^ cell expression detected at a lower level, while IFNγ expression was not detected at all (data not shown). In the mixed lymphocyte reactions (Fig. 3E), both IL-3 and IL-3/CSF-1 CD11c^+^ cells failed to efficiently stimulate allogeneic T cell proliferation, with much lower levels of percentages on proliferated T cells, in contrast to GM-CSF-derived DCs. These data support that IL-3 and IL-3/CSF-1-derived CD11c^+^ cells are macrophages with a possible regulatory phenotype. Although both express CD11c, CD11b, costimulatory molecules and MHC-class II (Fig. 3A), in contrast with the data reported in a previous study [9], we did not find any evidence to suggest that they have conventional myeloid DC properties. Hence, expression of these cell surface markers under specific cytokine conditions or in response to certain inflammatory cues is insufficient to classify a monocytic cell population as DCs [30].

## Conclusions

The ability of CSF-1 to modulate the IL-3-driven hematopoietic pathway may have implications in allergic inflammations. IL-3 is the major inducer of blood basophils and tissue mast cells, which are FcεRI^+^ effector cells that regulate immediate hypersensitivity. CSF-1 may on one hand mitigate overproduction of basophils and mast cells that can cause undesired tissue damage, while on the other hand mobilizing IL-10-producing monocytic cells that exert anti-inflammatory functions and/or facilitate the development of Th2 and humoral responses [31]. An alternative perspective is that, because CSF-1 drives CD11c^-^MHC-class II^-^ macrophage generation, IL-3 may function to divert from a wound healing (mediated by CSF-1 alone) to an immunomodulatory phenotype. Nevertheless, the ability of IL-3 together with CSF-1 to regulate CD11c^+^ macrophage expansion highlights the role of cytokine combination in plasticity of the BM progenitors that further contributes to the heterogeneity of the antigen-presenting cell system.

## Supporting Information

Figure S1 **Total cells are comparable in IL-3 and IL-3/CSF-1 cultures.** BM cells were isolated and cultured with IL-3, CSF-1 or IL-3/CSF-1 in triplicates in the 24 well plate. At days 3, 6, 9 and 12, total cells were collected and counted from each well. Cell numbers are presented as the mean ± SEM. Data shown are representative of four separate experiments.
(TIFF)

Figure S2 **CSF-1 does not enhance GMDC generation.** BM cells were isolated and cultured with GM-CSF and GM-CSF/CSF-1 in triplicates in a 24 well plate. At days 3, 6 and 9, total cells were numerated. Cells were labeled with fluorochrome-conjugated antibodies. (A) Live DCs from different culture conditions were identified as the CD11b+CD11c+ population in flow cytometry. (B) Total cell numbers, together with DC percentages and numbers, were determined as the mean ± SEM in the kinetic analysis. Data shown are representative of three separate experiments.
(TIFF)

Figure S3 **CSF1R-blocking does not impair generation of GMDCs.** BM cells were isolated and cultured with GM-CSF in the presence of AFS98 or the isotype control antibody in triplicates in a 24 well plate. At day 7, cells were collected and counted. Cells were labeled with fluorochrome-conjugated antibodies. (A) Representative dot plots from treated cultures were shown. (B) Percentages and numbers of CD11b^+^CD11c^+^ cells were determined by flow cytometry and presented as the mean ± SEM. Data shown are representative of three separate experiments.
(TIFF)

## References

[1] Niemeyer CM, Sieff CA, Mathey-Prevot B, Wimperis JZ, Bierer BE, et al (1989) Expression of human interleukin-3 (multi-CSF) is restricted to human lymphocytes and T-cell tumor lines. Blood 73: 945–951. 2645952

[2] Plaut M, Pierce JH, Watson CJ, Hanley-Hyde J, Nordan RP, et al (1989) Mast cell lines produce lymphokines in response to cross-linkage of Fc epsilon RI or to calcium ionophores. Nature 339: 64–67. 2469965 10.1038/339064a0

[3] Shen T, Kim S, Do JS, Wang L, Lantz C, et al (2008) T cell-derived IL-3 plays key role in parasite infection-induced basophil production but is dispensable for in vivo basophil survival. International immunology 20: 1201–1209. 18632726 10.1093/intimm/dxn077

[4] Aoki I, Tanaka S, Ishii N, Minami M, Klinman DM (1996) Contribution of interleukin-3 to antigen-induced Th2 cytokine production. European journal of immunology 26: 1388–1393. 8647221 10.1002/eji.1830260631

[5] Lantz CS, Boesiger J, Song CH, Mach N, Kobayashi T, et al (1998) Role for interleukin-3 in mast-cell and basophil development and in immunity to parasites. Nature 392: 90–93. 9510253 10.1038/32190

[6] Morris CF, Young IG, Hapel AJ (1990) Molecular and cellular biology of interleukin-3. Immunology series 49: 177–214. 2090251

[7] Hapel AJ, Fung MC, Johnson RM, Young IG, Johnson G, et al (1985) Biologic properties of molecularly cloned and expressed murine interleukin-3. Blood 65: 1453–1459. 3922456

[8] Metcalf D, Begley CG, Johnson GR, Nicola NA, Lopez AF, et al (1986) Effects of purified bacterially synthesized murine multi-CSF (IL-3) on hematopoiesis in normal adult mice. Blood 68: 46–57. 3087441

[9] Baumeister T, Rossner S, Pech G, de Bruijn MF, Leenen PJ, et al (2003) Interleukin-3Ralpha+ myeloid dendritic cells and mast cells develop simultaneously from different bone marrow precursors in cultures with interleukin-3. The Journal of investigative dermatology 121: 280–288. 12880419 10.1046/j.1523-1747.2003.12380.x

[10] Nakahata T, Kobayashi T, Ishiguro A, Tsuji K, Naganuma K, et al (1986) Extensive proliferation of mature connective-tissue type mast cells in vitro. Nature 324: 65–67. 3491321 10.1038/324065a0

[11] Morris CF, Salisbury J, Kobayashi M, Townsend PV, Hapel AJ (1990) Interleukin 3 alone does not support the proliferation of bone marrow cells from A/J mice: a novel system for studying the synergistic activities of IL-3. British journal of haematology 74: 131–137. 2138495 10.1111/j.1365-2141.1990.tb02555.x

[12] Stanley ER, Berg KL, Einstein DB, Lee PS, Pixley FJ, et al (1997) Biology and action of colony—stimulating factor-1. Molecular reproduction and development 46: 4–10. 8981357 10.1002/(SICI)1098-2795(199701)46:1<4::AID-MRD2>3.0.CO;2-V

[13] Chitu V, Stanley ER (2006) Colony-stimulating factor-1 in immunity and inflammation. Current opinion in immunology 18: 39–48. 16337366 10.1016/j.coi.2005.11.006

[14] Mossadegh-Keller N, Sarrazin S, Kandalla PK, Espinosa L, Stanley ER, et al (2013) M-CSF instructs myeloid lineage fate in single haematopoietic stem cells. Nature 497: 239–243. 23575636 10.1038/nature12026 PMC3679883

[15] Fancke B, Suter M, Hochrein H, O'Keeffe M (2008) M-CSF: a novel plasmacytoid and conventional dendritic cell poietin. Blood 111: 150–159. 17916748 10.1182/blood-2007-05-089292

[16] Zhang L, Friedman AD (2011) SHP2 tyrosine phosphatase stimulates CEBPA gene expression to mediate cytokine-dependent granulopoiesis. Blood 118: 2266–2274. 21725048 10.1182/blood-2011-01-331157 PMC3162355

[17] Jack GD, Zhang L, Friedman AD (2009) M-CSF elevates c-Fos and phospho-C/EBPalpha(S21) via ERK whereas G-CSF stimulates SHP2 phosphorylation in marrow progenitors to contribute to myeloid lineage specification. Blood 114: 2172–2180. 19587381 10.1182/blood-2008-11-191536 PMC2744575

[18] Caux C, Vanbervliet B, Massacrier C, Durand I, Banchereau J (1996) Interleukin-3 cooperates with tumor necrosis factor alpha for the development of human dendritic/Langerhans cells from cord blood CD34+ hematopoietic progenitor cells. Blood 87: 2376–2385. 8630401

[19] Kawano Y, Takaue Y, Hirao A, Abe T, Saito S, et al (1991) Synergistic effect of recombinant interferon-gamma and interleukin-3 on the growth of immature human hematopoietic progenitors. Blood 77: 2118–2121. 1903073

[20] Buelens C, Bartholome EJ, Amraoui Z, Boutriaux M, Salmon I, et al (2002) Interleukin-3 and interferon beta cooperate to induce differentiation of monocytes into dendritic cells with potent helper T-cell stimulatory properties. Blood 99: 993–998. 11807004 10.1182/blood.v99.3.993

[21] Ebner S, Hofer S, Nguyen VA, Furhapter C, Herold M, et al (2002) A novel role for IL-3: human monocytes cultured in the presence of IL-3 and IL-4 differentiate into dendritic cells that produce less IL-12 and shift Th cell responses toward a Th2 cytokine pattern. Journal of immunology 168: 6199–6207. 10.4049/jimmunol.168.12.6199 12055233

[22] Bartelmez SH, Sacca R, Stanley ER (1985) Lineage specific receptors used to identify a growth factor for developmentally early hemopoietic cells: assay of hemopoietin-2. Journal of cellular physiology 122: 362–369. 2981895 10.1002/jcp.1041220305

[23] Breen FN, Hume DA, Weidemann MJ (1990) The effects of interleukin 3 (IL-3) on cells responsive to macrophage colony-stimulating factor (CSF-1) in liquid murine bone marrow culture. British journal of haematology 74: 138–145. 2180468 10.1111/j.1365-2141.1990.tb02556.x

[24] Sasmono RT, Oceandy D, Pollard JW, Tong W, Pavli P, et al (2003) A macrophage colony-stimulating factor receptor-green fluorescent protein transgene is expressed throughout the mononuclear phagocyte system of the mouse. Blood 101: 1155–1163. 12393599 10.1182/blood-2002-02-0569

[25] Sheng KC, Kalkanidis M, Pouniotis DS, Wright MD, Pietersz GA, et al (2008) The adjuvanticity of a mannosylated antigen reveals TLR4 functionality essential for subset specialization and functional maturation of mouse dendritic cells. Journal of immunology 181: 2455–2464. 10.4049/jimmunol.181.4.2455 18684936

[26] Sudo T, Nishikawa S, Ogawa M, Kataoka H, Ohno N, et al (1995) Functional hierarchy of c-kit and c-fms in intramarrow production of CFU-M. Oncogene 11: 2469–2476. 8545103

[27] Greter M, Helft J, Chow A, Hashimoto D, Mortha A, et al (2012) GM-CSF controls nonlymphoid tissue dendritic cell homeostasis but is dispensable for the differentiation of inflammatory dendritic cells. Immunity 36: 1031–1046. 22749353 10.1016/j.immuni.2012.03.027 PMC3498051

[28] Nakamura T, Datta R, Kharbanda S, Kufe D (1991) Regulation of jun and fos gene expression in human monocytes by the macrophage colony-stimulating factor. Cell growth & differentiation: the molecular biology journal of the American Association for Cancer Research 2: 267–272. 1712226

[29] Reddy EP, Korapati A, Chaturvedi P, Rane S (2000) IL-3 signaling and the role of Src kinases, JAKs and STATs: a covert liaison unveiled. Oncogene 19: 2532–2547. 10851052 10.1038/sj.onc.1203594

[30] Drutman SB, Kendall JC, Trombetta ES (2012) Inflammatory spleen monocytes can upregulate CD11c expression without converting into dendritic cells. Journal of immunology 188: 3603–3610. 10.4049/jimmunol.1102741 PMC4594880 22442444

[31] Murray PJ, Wynn TA (2011) Protective and pathogenic functions of macrophage subsets. Nature reviews Immunology 11: 723–737. 10.1038/nri3073 PMC3422549 21997792

