# Supplementary figures and images for: IL-3 and CSF-1 Interact to Promote Generation of CD11c+ IL-10-Producing Macrophages
Source: PLoS One. 2014 Apr 17;9(4):e95208. doi: 10.1371/journal.pone.0095208 (PMC3990611; doi:10.1371/journal.pone.0095208)

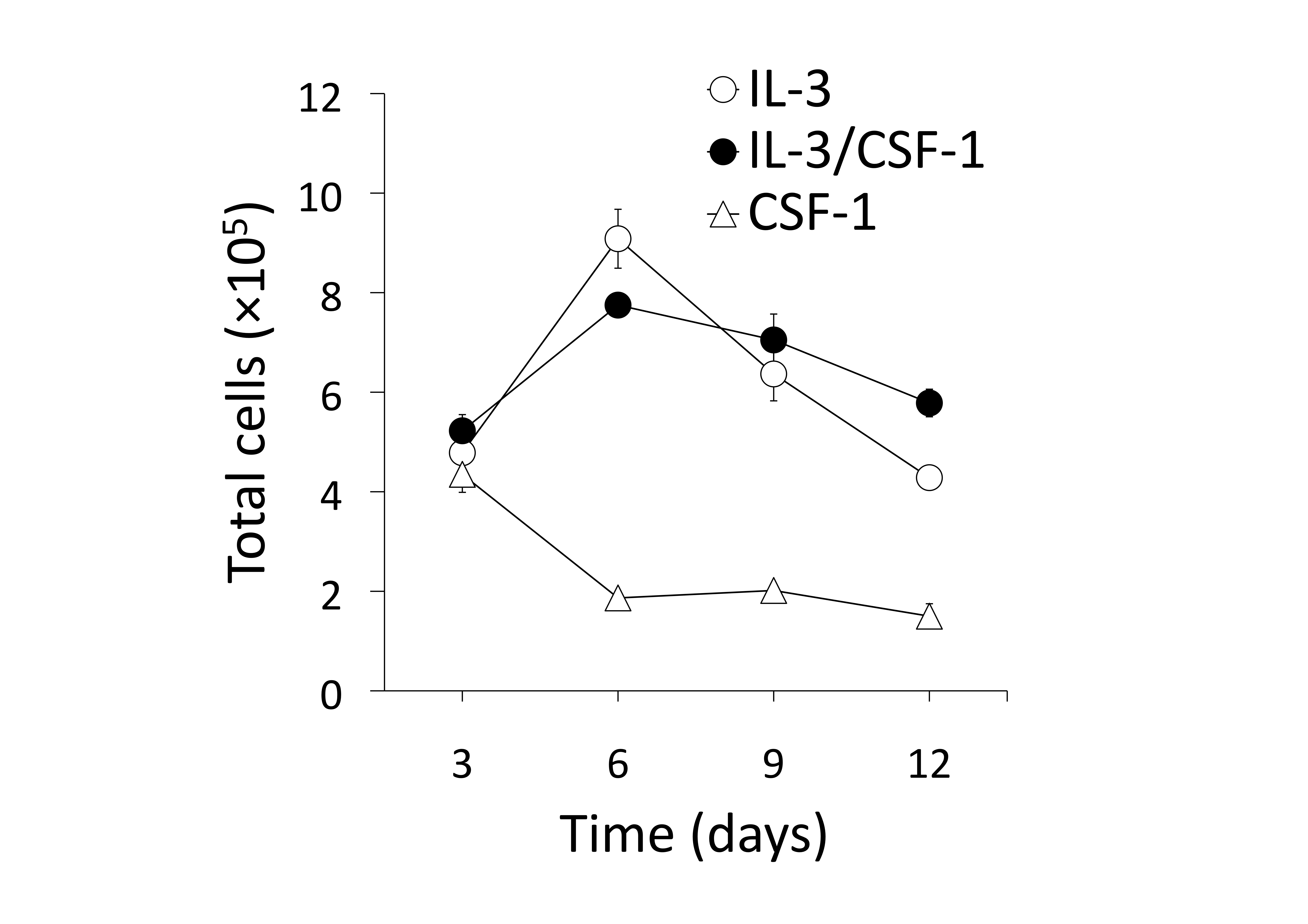

Supplement: Figure S1 — Total cells are comparable in IL-3 and IL-3/CSF-1 cultures. BM cells were isolated and cultured with IL-3, CSF-1 or IL-3/CSF-1 in triplicates in the 24 well plate. At days 3, 6, 9 and 12, total cells were collected and counted from each well. Cell numbers are presented as the mean ± SEM. Data shown are representative of four separate experiments. (TIFF) [file pone.0095208.s001.tiff]

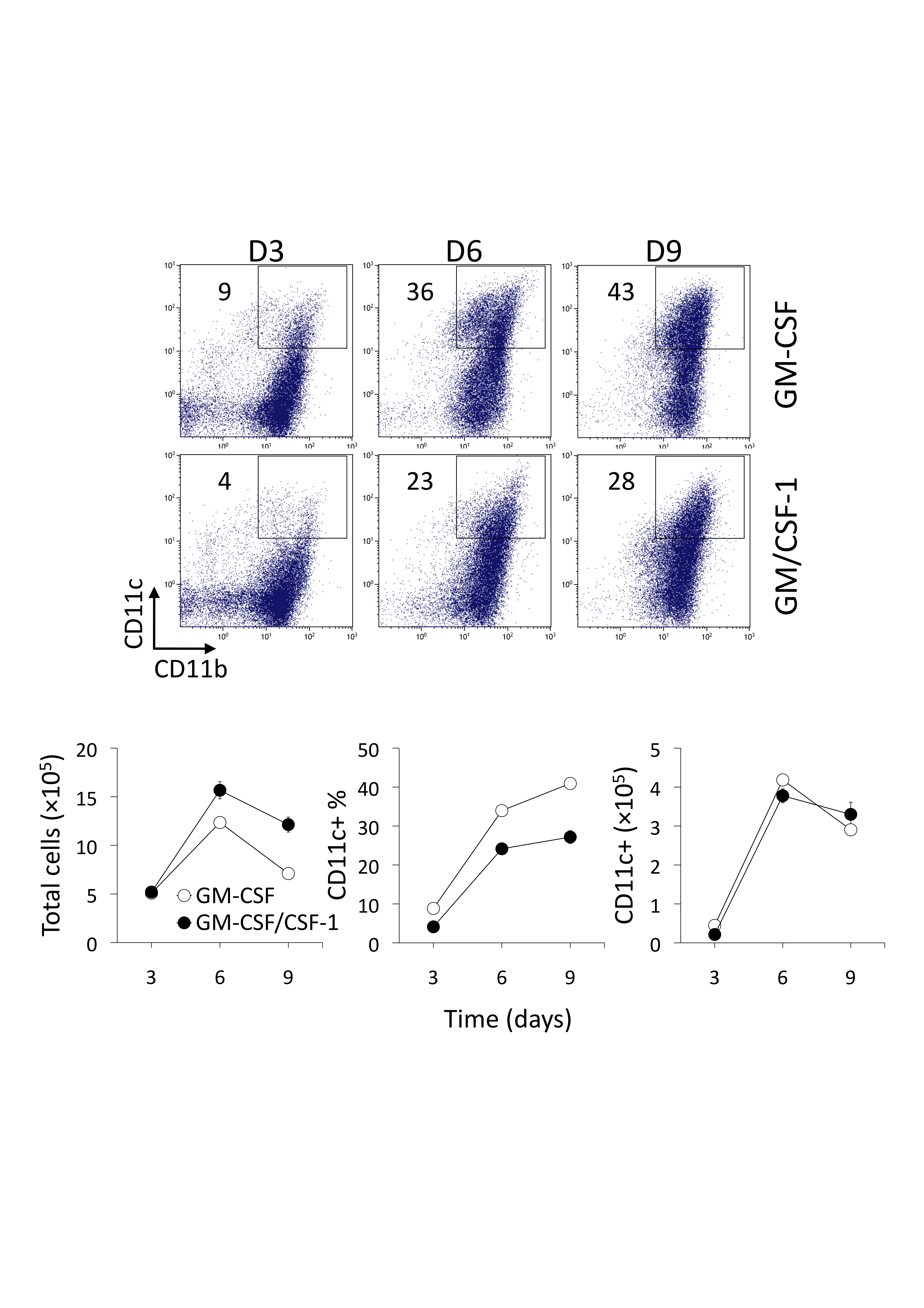

Supplement: Figure S2 — CSF-1 does not enhance GMDC generation. BM cells were isolated and cultured with GM-CSF and GM-CSF/CSF-1 in triplicates in a 24 well plate. At days 3, 6 and 9, total cells were numerated. Cells were labeled with fluorochrome-conjugated antibodies. (A) Live DCs from different culture conditions were identified as the CD11b+CD11c+ population in flow cytometry. (B) Total cell numbers, together with DC percentages and numbers, were determined as the mean ± SEM in the kinetic analysis. Data shown are representative of three separate experiments. (TIFF) [file pone.0095208.s002.tiff]

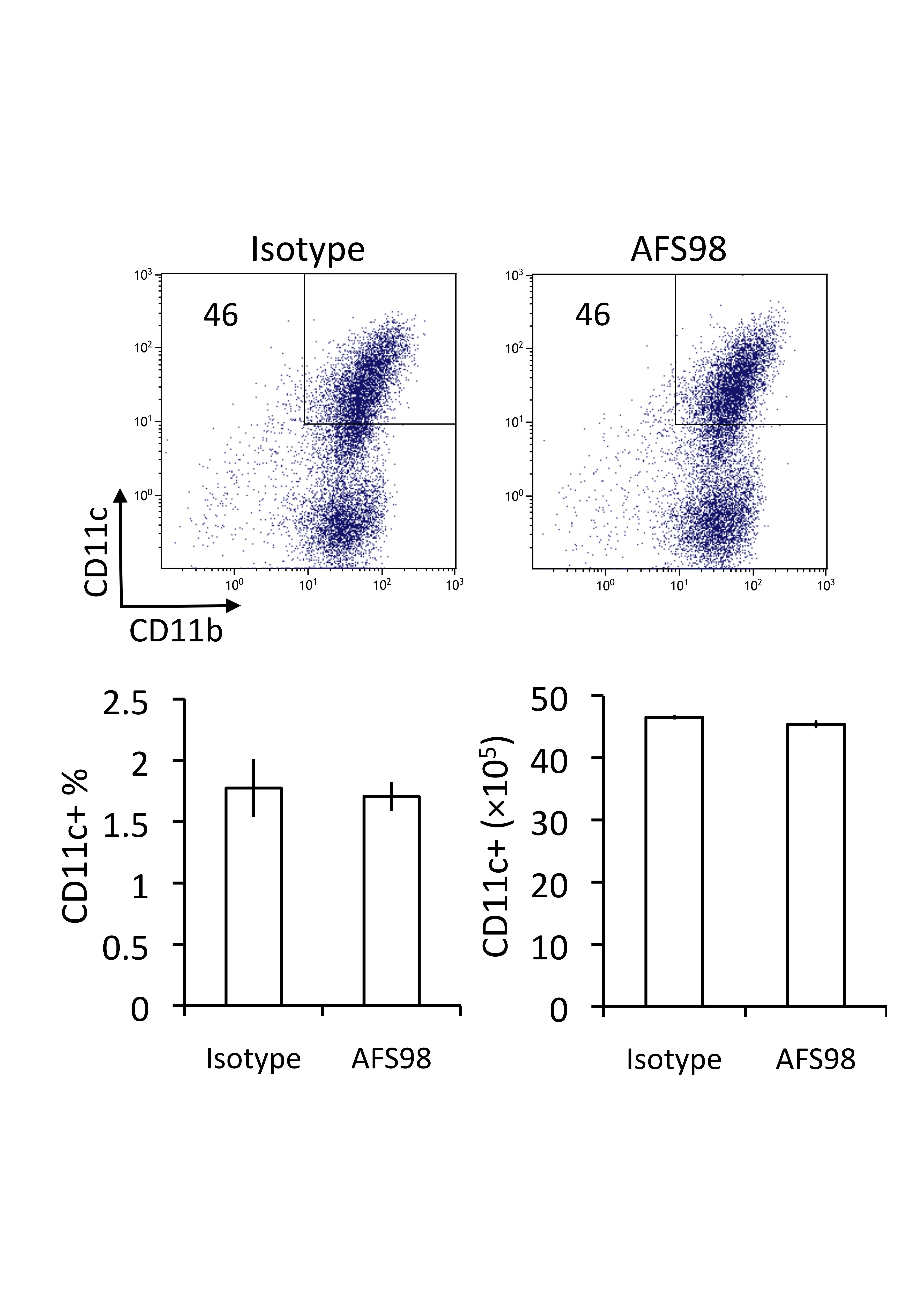

Supplement: Figure S3 — CSF1R-blocking does not impair generation of GMDCs. BM cells were isolated and cultured with GM-CSF in the presence of AFS98 or the isotype control antibody in triplicates in a 24 well plate. At day 7, cells were collected and counted. Cells were labeled with fluorochrome-conjugated antibodies. (A) Representative dot plots from treated cultures were shown. (B) Percentages and numbers of CD11b+CD11c+ cells were determined by flow cytometry and presented as the mean ± SEM. Data shown are representative of three separate experiments. (TIFF) [file pone.0095208.s003.tiff]
